# Cardiovascular toxicity of CDK4/6 inhibitors combined with endocrine therapy versus endocrine therapy alone in HR+/HER2- breast cancer: a real-world study based on the FAERS database

**DOI:** 10.3389/fphar.2026.1831639

**Published:** 2026-07-01

**Authors:** Shuai Ma, Lu Li, Yanwei Chen, Yongshun Zhao

**Affiliations:** 1 Department of Pharmacy, First Affiliated Hospital of Dalian Medical University, Dalian, China; 2 College of Pharmacy, Dalian Medical University, Dalian, China; 3 Department of Neurosurgery, First Affiliated Hospital of Dalian Medical University, Dalian, China

**Keywords:** cardiovascular adverse events, CDK 4/6 inhibitors, endocrine therapy, FAERS, pharmacovigilance

## Abstract

**Objective:**

This study was designed to examine the relationship between CDK4/6 inhibitor therapy and cardiovascular adverse events (CVAEs).

**Method:**

A disproportionality analysis was performed on reports from the FDA Adverse Event Reporting System (FAERS) database covering the period from 2015 to the first quarter of 2025. The reporting odds ratio (ROR) and information components (IC) were derived from this analysis. CVAEs were categorized into ten specific groups based on the standardized MedDRA queries (SMQs). Multifactorial logistic regression analysis was conducted to identify factors associated with CVAEs following treatment with CDK4/6 inhibitors.

**Results:**

Analysis of 4,002 CVAEs associated with CDK4/6 inhibitors indicated a stronger reporting association of cardiotoxicity for ribociclib (OR = 1.55, p < 0.001), especially arrhythmia. Ribociclib + fulvestrant showed a strong arrhythmia signal (ROR = 2.86; n = 188), while ribociclib + letrozole had the strongest QT prolongation signal (ROR = 6.51; n = 291) and an increased reporting signal for shock (ROR = 4.33). Abemaciclib + fulvestrant exhibited stronger thrombotic signals (ROR = 2.50) than with letrozole (ROR = 1.24). Patients over 65 showed stronger CVAE signals (OR = 1.29, p < 0.001), further elevated by letrozole/fulvestrant combinations (OR = 1.83/1.32, p < 0.001). Most CVAEs occurred early (0–60 days), with peak incidence for ribociclib + letrozole at 0–30 days and late arrhythmia resurgence for ribociclib + fulvestrant (210–240 days). Shock-related mortality was the most fatal outcome; thrombotic events prolonged hospitalization.

**Conclusion:**

CDK4/6 inhibitor combinations show notable cardiovascular reporting signals, with more frequent CVAE reports in letrozole-containing regimens. These signals suggest enhanced vigilance for thrombosis, arrhythmias, QT prolongation, and shock may be warranted. Cohort and long-term trials are needed to validate these safety findings.

## 1 Introduction

Breast cancer (BC) is the most prevalent malignant tumor among women worldwide. The 2022 Global Cancer Statistics Report indicates approximately 2.3 million new BC cases, representing 11.6% of global cancer diagnoses (Bardia et al., 2023; Bray et al., 2024). The most common subtype, accounting for approximately 70% of all cases, is hormone receptor-positive (HR+) and human epidermal growth factor receptor-negative (HER2-) BC, which can be classified into Luminal A type and Luminal B type (HER2-) (Geisler et al., 2023; Huang et al., 2024; Martins et al., 2023; Loibl et al., 2021). HR+/HER2- BC is estrogen-driven (Bardia and Hurvitz, 2018). Endocrine therapy (ET) suppresses tumor growth by targeting estrogen signaling (Hanker et al., 2020), but resistance frequently develops (Hoffmann et al., 2004).

As a milestone therapeutic strategy, combination regimens of three approved CDK4/6 inhibitors (palbociclib, abemaciclib, ribociclib) plus ET restrain cyclin D–CDK complex activity, inhibit retinoblastoma (Rb) protein phosphorylation and induce G_1_ cell-cycle arrest, substantially reshaping the treatment paradigm for advanced HR+/HER2− BC (Fountzilas et al., 2020; Fontanella et al., 2022; Braal et al., 2021). Palbociclib gained the first US FDA approval in 2015 and pioneered the clinical application of this drug class (Fradley et al., 2023; Roberto et al., 2021). Existing network meta-analyses have validated that pairing CDK4/6 inhibitors with ET yields superior progression-free survival (PFS) compared with single-agent ET (Liu et al., 2023).

However, combination treatment is accompanied by distinct adverse reactions, including hematologic toxicity (neutropenia, anemia), lymphedema, gastrointestinal discomfort (diarrhea, nausea), fatigue and alopecia (Spring et al., 2017; Pavlovic et al., 2023; Iwata, 2018). Cardiovascular adverse events (CVAEs) attract growing clinical attention owing to subtype-specific safety risks: ribociclib confers a 6.5% risk of QT interval prolongation (Slamon et al., 2018; Lu et al., 2024), abemaciclib bears official warnings for venous thromboembolism (VTE), and the PALOMA-3 trial recorded a 2% incidence of thromboembolic events under palbociclib therapy (Di Cosimo et al., 2023; Verma et al., 2016).

While disproportionality analyses report CDK4/6 inhibitor-associated cardiovascular adverse events (CVAEs) (Fradley et al., 2023; Li et al., 2025; Kim, 2024), critical gaps remain: differences in CVAEs between CDK4/6 inhibitors combined with aromatase inhibitors (AIs: letrozole, anastrozole, exemestane)/fulvestrant, and comparative CVAE profiles of combination therapy versus AI/fulvestrant monotherapy. To fill these gaps, we used the FDA Adverse Event Reporting System (FAERS) to perform a multidimensional analysis of cardiovascular adverse events across CDK4/6 inhibitor combinations with AI or fulvestrant, and assessed corresponding reporting associations. The results may help refine safety management for advanced HR+/HER2- BC.

## 2 Methods

### 2.1 Data source

The FAERS is a public, free post-market safety monitoring system that collects spontaneous adverse event reports submitted by healthcare professionals (e.g., physicians, pharmacists, nurses) and consumers (e.g., patients, family members, legal representatives) worldwide. The system codes AEs into preferred terms (PTs) and system organ classes (SOCs) based on the medical dictionary for regulatory activities (MedDRA), with each PT mapped to its corresponding SOC.

To enhance analytical specificity, PTs are systematically categorized into Standardized MedDRA Queries (SMQs) to define specific medically important events. SMQs were selected to balance broad capture and clinical relevance. Broad SMQs maximize sensitivity by including all related PTs, ensuring no potential CVAE signals are missed in spontaneous reports. In contrast, narrow definitions risk excluding relevant events and underestimating true reporting patterns. However, broad SMQs aggregate heterogeneous PTs (e.g., embolic and thrombotic events), which may reduce outcome specificity.

This pharmacovigilance analysis employed the FAERS database (Q1 2015-Q1 2025) to retrospectively assess CDK4/6 inhibitor-related CVAEs (Gomez-Lumbreras et al., 2023; Abou Kaoud et al., 2023).

### 2.2 Procedures

**FIGURE 1:**
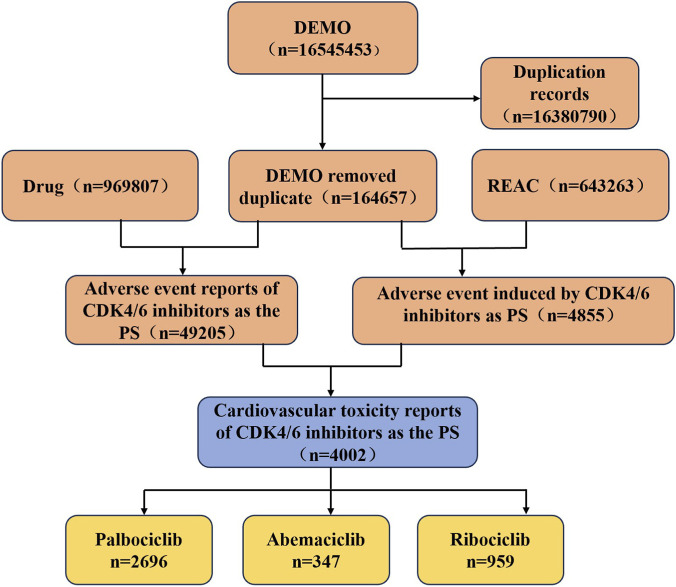
Process of selecting cases of CDK4/6 inhibitors-associated CV from the FAERS database. Abbreviations: DEMO, demographic and administrative information; REAC, reactions; n: the number of reports; PS, primary suspect.

Adverse events were coded via MedDRA (v26.1) using 10 SMQs (e.g., cardiac failure, QT prolongation) (Supplementary Tables S1, S2). FAERS reports of CDK4/6 inhibitors combined with fulvestrant/AIs (letrozole/anastrozole/exemestane) were analyzed, with drugs as “primary suspect” (PS). Duplicates were excluded per FDA standards: identical CASEID retained latest FDA_DT; matching CASEID/FDA_DT kept highest PRIMARY_ID (Figure 1) (Ren et al., 2022).

### 2.3 Statistical analysis

Disproportionality Analysis (also known as case/non-case analysis) evaluates the relative weighting of specific drug-adverse event associations using a 2 × 2 contingency table (Table 1) with a moving scale approach, which is widely applied in pharmacovigilance research. Two signal detection metrics were used in this work: the reporting odds ratio (ROR) (Equation 1) and information component (IC) (Equation 2). These two indicators were applied to identify potential links between study drugs and adverse events, and their corresponding calculation formulas are presented below.

$$ROR=\left(a/b\right)/\left(c/d\right)$$ (1)

$$\text{IC}=\log_{2}\frac{a\left(a+b+c+d\right)}{\left(a+b\right)\left(a+c\right)}$$ (2)

**TABLE 1:** Two-by-two contingency (2 × 2) table for disproportionality analyses.

Types of drugs	Target adverse events	Other adverse events
Target drug	a	b
All other drugs	c	d

Furthermore, the formulas for calculating the 95% confidence interval (CI) for the ROR (Equation 3) and IC (Equation 4) are as follows:

$$95\%CI\;of\;ROR=e^{\ln\left(ROR\right)\pm 1.96\left(1/a+1/b+1/c+1/d\right){\text{​}}^{0.5}}$$ (3)

$$95\%\text{CI of IC}=e^{In\left(IC\right)\pm 1.96\sqrt{\left(\frac{1}{a}+\frac{1}{b}+\frac{1}{c}+\frac{1}{d}\right)}}$$ (4)

A potential signal was deemed statistically significant if the lower bound of the 95% CI for the ROR >1 (ROR025 > 1). For IC, statistical significance is considered when the lower limit of the 95% CI > 0 (IC025 > 0) (Zhang et al., 2024).

Multivariable logistic regression was used to estimate adjusted odds ratios (ORs) for CVAEs reporting among patients receiving CDK4/6 inhibitors, with statistical significance set at a two-tailed p-value <0.05. All regression analyses drew on all FAERS reports documenting CDK4/6 inhibitor use. CVAE occurrence was coded as a binary outcome (event present or absent) for every eligible record, rather than restricting the dataset only to cases that reported CVAEs. Basic demographic covariates including age, sex, and body weight were adjusted in the regression models. Prior to multivariable modeling, univariate logistic regression was performed to screen candidate variables, retaining only factors with significant univariate correlations (p < 0.05) in the final model. This analytical method was also applied to evaluate SMQ-defined hospitalization and fatal outcomes related to CVAEs. All independent variables, including demographic characteristics and SMQ-based event metrics, were clearly defined and standardized before modeling. Consistent with FAERS database characteristics, records with missing covariate data were excluded without imputation. Variance inflation factor (VIF) analysis was used to detect multicollinearity, and variables with VIF >10 were excluded to avoid collinearity bias. The Hosmer–Lemeshow test was utilized to assess model fitness; distinct from the conventional association significance threshold, a Hosmer–Lemeshow p-value >0.05 signifies good model fit, indicating consistency between observed and predicted outcomes. Univariate analysis identified ten SMQs associated with CVAEs, and statistically significant SMQs were included in the multivariable model to determine independent predictors of CVAE-related hospitalization. All regression analyses drew on all FAERS reports documenting CDK4/6 inhibitor use. CVAE occurrence was coded as a binary outcome (event present or absent) for every eligible record, rather than restricting the dataset only to cases that reported CVAEs.

Notably, ROR and IC were computed for disproportionality signal detection using a case-noncase design, whereas adjusted OR from multivariate logistic regression quantified covariate-corrected reporting associations. Given their distinct computational frameworks and statistical interpretations, these metrics are not interchangeable.

These analyses are exploratory and reflect reporting associations only, not causal relationships.

All statistical analyses were conducted using R 4.2.1 software.

## 3 Results

### 3.1 Descriptive analysis

**FIGURE 2:**
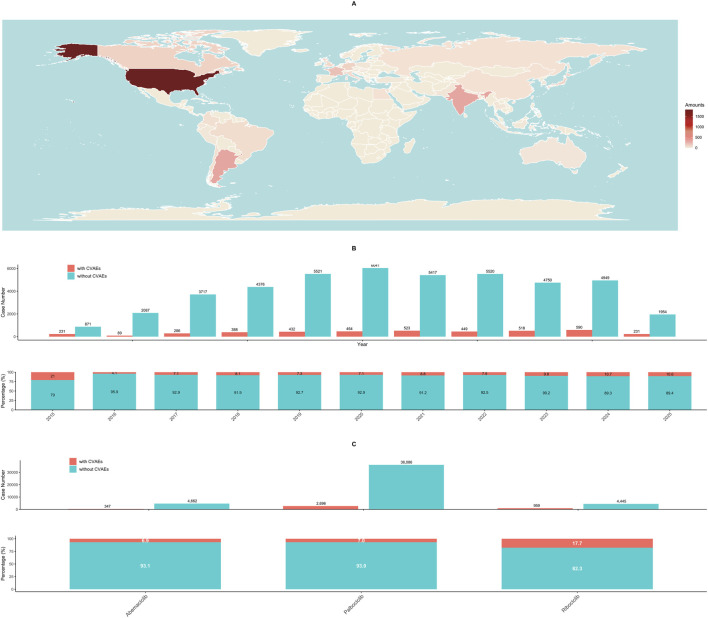
Scanning for CDK4/6 inhibitor-related CVAEs based on the FAERS database. **(A)** world map illustrating the distribution of cardiovascular adverse events associated with the use of CDK4/6 inhibitors across different countries, as recorded in the FAERS database. **(B)** the upper bar chart illustrates the annual number of CDK4/6 inhibitor reports, both with and without CVAEs, over a period of 11 years. The stacked bar chart below shows the annual proportions of CDK4/6 inhibitor reports with and without CVAEs. **(C)** the upper bar chart displays the number of reports with and without CVAEs for various CDK4/6 inhibitors. The proportional bar chart below illustrates the proportions of reports with and without CVAEs for these inhibitors.

From 2015 to 2025, FAERS data identified 49,205 CDK4/6 inhibitor-related adverse events, with 4,002 CVAE cases (Figure 2). The number of CVAE reports increased continuously over the study period, with their proportion climbing from 3% in 2015 to 14.7% in 2024. Stratified by agent, CVAE cases numbered 2,696 for palbociclib, 959 for ribociclib and 347 for abemaciclib. Female patients predominated (98.9%), with 58.7% aged ≥65 years versus 41.3% under 65. Healthcare professionals submitted 89.1% reports. Serious outcomes included hospitalization (37.4%) and death (13.9%). Reports mainly originated from the US (45.6%), Argentina (7.9%), and India (8.0%) (Table 2).

**TABLE 2:** Characteristics of patients with CVAEs associated with CDK4/6 inhibitors in the FAERS database (1 January 2015 to 31 March 2025).

Characteristics	CDK4/6 inhibitors	Palbociclib	Abemaciclib	Ribociclib
Target-pt report	4,002	2,696	347	959
Sex, n (%)	​	​	​	​
Female	3,956 (98.9)	2,666 (98.9)	342 (98.6)	948 (98.9)
Male	46 (1.1)	30 (1.1)	5 (1.4)	11 (1.1)
Age, n (%)	​	​	​	​
18 to <65	1,651 (41.3)	979 (36.3)	167 (48.1)	505 (52.7)
65 to <85	2,351 (58.7)	1,717 (63.7)	180 (51.9)	454 (47.3)
Outcome, n (%)	​	​	​	​
Non-serious outcome	194 (4.8)	156 (5.8)	24 (6.9)	14 (1.5)
Serious outcome	3,808 (95.2)	2,536 (94.2)	323 (93.1)	​
Death	557 (13.9)	330 (12.2)	45 (13)	182 (19.0)
Lift-threatening	158 (3.9)	72 (2.7)	15 (4.3)	71 (7.4)
Hospitalization	1,498 (37.4)	1,042 (38.6)	161 (46.4)	295 (30.8)
Disability	48 (1.1)	10 (0.4)	2 (0.6)	11 (1.1)
Other serious	1,565 (39.1)	1,082 (40.1)	100 (28.8)	383 (39.9)
Reporters, n (%)	​	​	​	​
Health professional	3,567 (89.1)	2,322 (86.1)	334 (96.2)	911 (94.9)
Non-health professional	63 (1.6)	40 (1.5)	9 (2.6)	14 (1.5)
Others	372 (9.3)	334 (12.4)	4 (1.2)	34 (3.5)
Reporting countries, n (%)	​	​	​	​
America (USA)	1,824 (45.6)	1,607 (59.6)	129 (37.2)	88 (9.2)
China	34 (0.8)	10 (0.4)	24 (6.9)	0 (0)
Japan	94 (2.3)	31 (1.1)	63 (18.2)	0 (0)
France	180 (4.5)	118 (4.4)	20 (5.8)	42 (4.4)
Argentina	315 (7.9)	261 (9.7)	67 (2.0)	47 (4.9)
Canada	107 (2.7)	73 (2.7)	3 (0.9)	31 (3.2)
India	319 (8.0)	312 (11.6)	2 (0.6)	5 (0.5)
Reporting year, n (%)	​	​	​	​
2015 (Q1-Q4)	32 (0.8)	32 (1.2)	0 (0)	0 (0)
2016	89 (2.2)	89 (3.3)	0 (0)	0 (0)
2017	286 (7.1)	273 (10.1)	0 (0)	13 (1.2)
2018	388 (9.7)	306 (11.3)	5 (1.4)	77 (7.4)
2019	432 (10.8)	337 (12.5)	23 (6.5)	72 (6.9)
2020	464 (11.6)	313 (11.6)	39 (11.0)	112 (10.7)
2021	523 (13.1)	350 (13.0)	40 (11.3)	133 (12.8)
2022	449 (11.2)	271 (10.1)	45 (12.7)	133 (12.8)
2023	518 (12.9)	263 (9.7)	66 (18.7)	189 (18.1)
2024	590 (14.7)	309 (11.5)	99 (28.0)	182 (17.5)
2025 (Q1)	231 (5.8)	153 (5.7)	30 (8.5)	48 (4.6)

Abbreviations: Q1, january to march; Q4, october to december; n, the number of reports; Target-pt report, Target preferred term report.

### 3.2 Disproportionality analysis

#### 3.2.1 Comparison of CDK4/6 inhibitor drugs associated with CVAEs

In CDK4/6 inhibitor therapy, the signal intensity for CVAEs was observed only with ribociclib (ROR = 1.07, 95% CI: 1.01–1.14) based on the ROR calculation method. In contrast, neither palbociclib (ROR = 0.52, 95% CI: 0.50–0.54) nor abemaciclib (ROR = 0.83, 95%CI: 0.75–0.91) showed statistically significant signals for CVAEs. Additionally, no significant signal was observed for CVAEs using the CI calculation method (Table 3).

**TABLE 3:** Disproportionality analysis calculated using ROR and IC.

Therapy drug	ROR	ROR025	ROR975	IC	IC025
CDK4/6 inhibitors	0.60	0.58	0.61	−0.52	−0.57
Palbociclib	0.52	0.50	0.54	−0.74	−0.79
Abemaciclib	0.83	0.75	0.91	−0.26	−0.4
Ribociclib	1.07	1.01	1.14	0.09	0.01

Abbreviations: ROR, reporting odds ratio; ROR_025_, the lower end of the 95% confidence interval of ROR; ROR_975_, the Upper end of the 95% confidence interval of ROR; IC, information component; the IC025, the lower end of the 95% confidence interval of IC.

#### 3.2.2 Spectrum of CVAEs associated with CDK4/6 inhibitors in HR+/HER2− BC treatment: SMQ-based analysis

**FIGURE 3:**
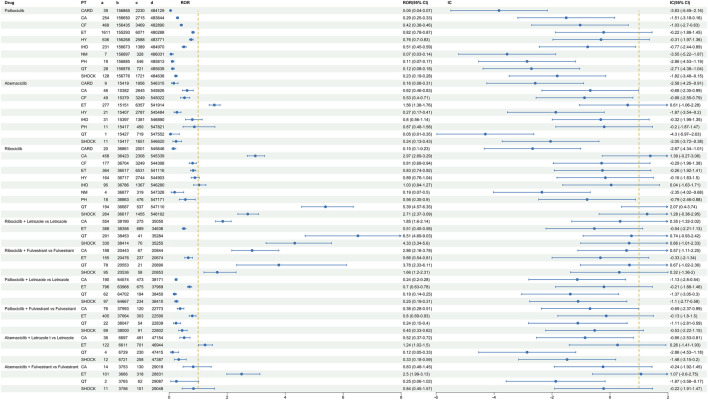
Disproportionality analyses calculated using ROR and IC. Disproportionality calculations using ROR and IC indicated elevated reporting signals of cardiovascular adverse events linked to CDK4/6 inhibitor monotherapy and combined regimens. Disproportionality analyses were performed using the reporting odds ratio (ROR) and information component (IC). These analyses identified disproportional reporting signals for cardiovascular adverse events associated with CDK4/6 inhibitors and their combination regimens. Abbreviations: CA, cardiac arrhythmias CARD, cardiomyopathy; CF, cardiac failure; ET, embolic and thrombotic events; HY, hypertension; IHD, ischemic heart disease; NM, noninfectious myocarditis; PH, pulmonary hypertension; QT, QT prolongation; ROR, reporting odds ratio; IC, information component.

Disproportionality analyses at the SOC level detected CDK4/6 inhibitor signals across 28 organ systems, with the strongest signal observed (n = 28,924; ROR = 2.00, 95%CI:1.97–2.04) (Table 4). General disorders and gastrointestinal conditions were the most frequently reported, with 34,357 and 26,946 cases respectively. While no meaningful SOC-level signal was found for cardiac disorders, subsequent SMQ analysis revealed evident differences in CVAE profiles among individual agents. Palbociclib showed no significant reporting signals for any cardiovascular SMQ. In contrast, abemaciclib was associated only with thrombosis and embolism events (ROR = 1.56, 95%CI: 1.38–1.76). For ribociclib, significant disproportional signals were observed for QT prolongation (ROR = 5.39, 95%CI: 4.57–6.35), cardiac arrhythmias (ROR = 2.97, 95%CI: 2.69–3.29), and shock (ROR = 2.71, 95%CI: 2.37–3.09). Collectively, four of the 10 cardiovascular SMQs exhibited statistically significant signals (Figure 3).

**TABLE 4:** Disproportionality analysis results for CDK4/6 inhibitors associated adverse events at the SOC level.

System organ class (SOC)	N	ROR	ROR_025_	ROR_975_	IC	IC_025_
Metabolism and utrition disorders	5,290	0.94	0.91	0.97	−0.06	−0.1
Renal and urinary disorders	2,190	0.84	0.8	0.88	−0.17	−0.24
Infections and infestations	9,302	1.06	1.03	1.09	0.05	0.02
Vascular disorders	3,675	0.84	0.81	0.87	−0.17	−0.22
Investigations	28,924	2	1.97	2.04	0.54	0.52
General disorders and administration site conditions	34,357	1.29	1.28	1.31	0.21	0.19
Gastrointestinal disorders	26,946	1.37	1.35	1.4	0.26	0.24
Respiratory, thoracic and mediastinal disorders	11,561	1.02	0.99	1.04	0.02	−0.02
Cardiac disorders	2077	0.41	0.39	0.43	−0.96	−1.03
Neoplasms benign, malignant and unspecified (incl cysts and polyps)	10,547	0.92	0.9	0.94	−0.08	−0.11
Blood and lymphatic system disorders	9,245	0.78	0.76	0.8	−0.24	−0.27
Skin and subcutaneous tissue disorders	12,433	0.39	0.39	0.4	−0.92	−0.95
Hepatobiliary disorders	2,135	0.53	0.5	0.55	−0.67	−0.74
Rvous system disorders	13,419	1.11	1.08	1.13	0.09	0.06
Psychiatric disorders	6,282	0.6	0.58	0.62	−0.51	−0.55
Musculoskeletal and connective tissue disorders	10,975	1.11	1.09	1.14	0.1	0.06
Eye disorders	2,818	0.95	0.91	1	−0.05	−0.11
Injury, poisoning and procedural complications	10,757	1.21	1.18	1.24	0.17	0.14
Surgical and medical procedures	1,576	1.4	1.32	1.5	0.31	0.22
Reproductive system and breast disorders	970	0.84	0.78	0.9	−0.18	−0.28
Ear and labyrinth disorders	978	1.68	1.55	1.83	0.46	0.35
Immune system disorders	1831	1.16	1.09	1.23	0.14	0.06
Endocrine disorders	234	0.26	0.22	0.29	−1.56	−1.76
Social circumstances	320	0.23	0.2	0.26	−1.7	−1.87
Product issues	303	0.49	0.43	0.55	−0.77	−0.95
Congenital, familial and genetic disorders	84	0.33	0.27	0.42	−1.23	−1.56
Pregnancy, puerperium and perinatal conditions	9	0.07	0.03	0.13	−3.36	−4.29

**FIGURE 4:**
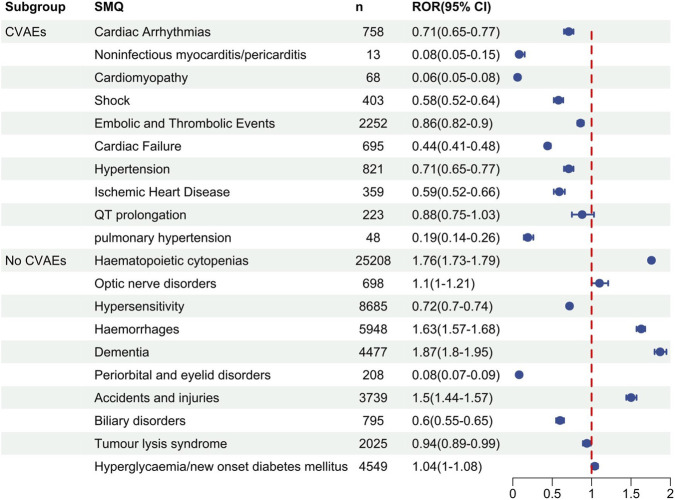
Disproportional analysis of SMQs in the cardiovascular system and other systems. Abbreviations: CVAEs, cardiovascular adverse events; SMQ, Standardized MedDRA Queries; n, the number of reports; ROR, reporting odds ratio; 95%CI, the 95% confidence interval.

Further analysis was performed based on significant and meaningful non-CVAE PTs (Table 5). Significant safety signals were identified in Figure 4 for the following SMQs: dementia (n = 4,477; ROR = 1.87 [95% CI: 1.80–1.95]), haematopoietic cytopenia (n = 25,208; ROR = 1.76 [1.73–1.79]), haemorrhages (n = 5,948; ROR = 1.63 [1.57–1.68]), accidents and injuries (n = 3,739; ROR = 1.50 [1.44–1.57]), optic nerve disorders (n = 698; ROR = 1.10 [1.00–1.21]), and hyperglycaemia/new onset diabetes mellitus (n = 4,549; ROR = 1.04 [1.00–1.08]).

**TABLE 5:** Top 20 Non-CVAEs PTs with the strongest signal strength for CDK4/6 inhibitors.

PT	a	ROR	ROR025	IC	IC025
Cardiac operation	11	22.82	2.95	1.49	0.34
Full blood count abnormal	1,355	22.63	18.84	1.49	1.38
Eye operation	21	21.78	5.11	1.49	0.64
Knee operation	20	20.75	4.85	1.48	0.61
Vitamin D increased	10	20.74	2.66	1.48	0.28
Allergic sinusitis	8	16.6	2.08	1.45	0.12
Full blood count decreased	1,156	16.07	13.55	1.44	1.33
Neutrophil percentage decreased	46	15.91	6.79	1.44	0.87
Product packaging difficult to open	30	15.56	5.48	1.44	0.73
Vein rupture	22	15.21	4.55	1.44	0.61
Snoring	7	14.52	1.79	1.43	0.03
Lithiasis	7	14.52	1.79	1.43	0.03
Poor quality product administered	81	14.01	7.64	1.42	0.98
Lymphocyte percentage increased	36	12.45	5.24	1.4	0.75
Yawning	6	12.45	1.5	1.4	−0.1
Immature granulocyte count increased	6	12.45	1.5	1.4	−0.1
Pre-existing condition improved	41	12.15	5.45	1.39	0.79
Red blood cell count abnormal	52	11.99	5.91	1.39	0.85
Lip exfoliation	17	11.76	3.44	1.39	0.46
Product dose omission in error	285	10.96	8.19	1.37	1.14
Dementia Alzheimer’s type	30	10.37	4.32	1.36	0.65
Rouleaux formation	15	10.37	3	1.36	0.38

Abbreviations: PT, preferred terms; a, the number; ROR, the reporting odds ratio; ROR025, the lower limit of the 95% confidence interval; IC, information components; IC025, the lower limit of the 95% confidence interval.

#### 3.2.3 The correlation between CVAEs and non-CVAEs

**FIGURE 5:**
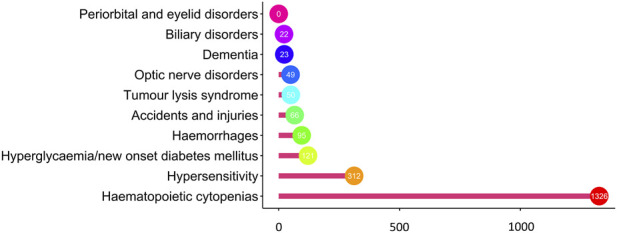
The lollipop plot to display the co-occurrence of CVAEs and non-CVAE SMQ.

We analyzed the co-occurrence of CVAEs with non-CVAEs at the SMQ level (Figure 5). The top three non-CVAE categories most frequently combined with CVAEs were haematopoietic cytopenias (n = 1,326), followed by hypersensitivity (n = 312), hyperglycaemia/new onset diabetes mellitus (n = 121), haemorrhages (n = 95), accidents and injuries (n = 66), tumour lysis syndrome (n = 55) and optic nerve disorders (n = 49).

#### 3.2.4 CVAEs by preferred term in patients receiving CDK4/6 inhibitors for HR+/HER2− BC

**FIGURE 6:**
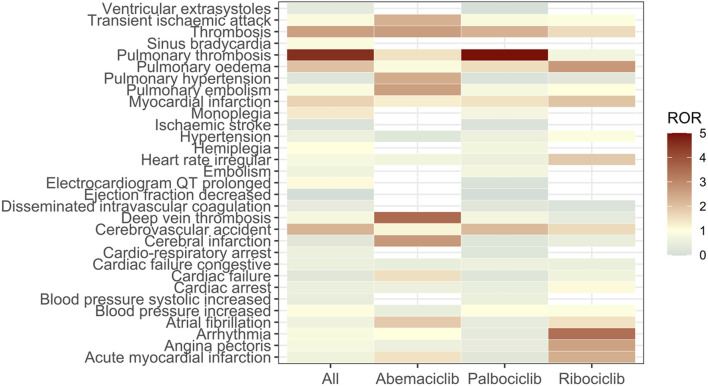
Heatmap of ROR values for the top 30 cardiovascular adverse events with ≥3 reported cases. Data are stratified by CDK4/6 inhibitor treatment groups (overall cohort, palbociclib, abemaciclib and ribociclib). All, CDK4/6 inhibitors.

At the PT level, we analyzed all cardiovascular adverse event reports linked to CDK4/6 inhibitors, focusing on the top 30 PTs with at least three reports. Among these, seven PTs showed positive signals (ROR025 > 1). We further examined reporting patterns separately for each agent. Ribociclib showed signals across 14 PTs, abemaciclib across 5 PTs, and palbociclib across 6 PTs. Notably, thrombosis, cerebrovascular accidents, and pulmonary thrombosis all produced positive signals for all three drugs, with thrombosis exhibiting the most prominent signal (Figure 6).

For abemaciclib, the most commonly reported PTs were pulmonary embolism (n = 64, ROR = 2.06, 95% CI: 1.6–2.66), thrombosis (n = 45, ROR = 2.25, 95% CI: 1.67–3.05), deep vein thrombosis (n = 29, ROR = 2.44, 95% CI: 1.68–3.56), cardiac failure (n = 24, ROR = 0.86, 95% CI: 0.57–1.29), and atrial fibrillation (n = 21, ROR = 1.25, 95% CI: 0.81–1.93).

In the case of palbociclib, the prominent adverse event signals at the PT level included thrombosis (n = 351, ROR 2.12, 95% CI: 1.85–2.42), cerebrovascular accident (n = 301, ROR = 2.17, 95% CI: 1.87–2.52), hypertension (n = 287, ROR = 0.6, 95% CI: 0.52–0.68), blood pressure increased (n = 275, ROR = 1.22, 95% CI: 1.06–1.41), and pulmonary embolism (n = 267, ROR = 0.76, 95% CI: 0.66–0.86).

For ribociclib, the five most common PTs were prolonged electrocardiogram QT interval (n = 179, ROR = 6.18, 95% CI: 5.19–7.36), pulmonary oedema (n = 109, ROR = 2.68, 95% CI: 2.18–3.28), hypertension (n = 101, ROR = 0.98, 95% CI: 0.80–1.20), thrombosis (n = 68, ROR = 1.40, 95% CI: 1.09–1.80), and pulmonary embolism (n = 65, ROR = 0.84, 95% CI: 0.65–1.08).

#### 3.2.5 Differences in CVAEs reporting signals between CDK4/6 inhibitors in combination with letrozole versus fulvestrant

The combination of ribociclib and fulvestrant yielded 188 reports of cardiac arrhythmia, accounting for 25.34% of relevant cases, and presented a strong reporting signal (ROR = 2.86, 95% CI: 2.16–3.78). By contrast, ribociclib plus letrozole had more arrhythmia reports (n = 554, 74.66%) but a weaker signal (ROR = 1.85, 95% CI: 1.60–2.14).

For QT prolongation, reports for ribociclib combined with fulvestrant were fewer (n = 78, 21.14%), with a ROR of 3.78 (95% CI: 2.33–6.11). The regimen of ribociclib plus letrozole contributed 78.86% of all cases (n = 291) and showed a markedly higher signal (ROR = 6.51, 95% CI: 4.69–9.03).

Similar trends were observed for shock. Ribociclib plus fulvestrant was linked to 22.35% of reports (n = 95) and a moderate signal (ROR = 1.66, 95% CI: 1.20–2.31). Ribociclib combined with letrozole made up 77.65% of cases (n = 330) and produced a stronger reporting signal (ROR = 4.33, 95% CI: 3.34–5.60).

Regarding thrombotic events, abemaciclib combined with fulvestrant accounted for 45.29% of all relevant reports (n = 101) and presented a prominent reporting signal (ROR = 2.50, 95% CI: 1.99–3.13). By comparison, abemaciclib plus letrozole yielded more reports (n = 122, 54.71%) but a weaker signal (ROR = 1.24, 95% CI: 1.02–1.50) (Figure 3).

### 3.3 Logistic regression analysis

#### 3.3.1 An investigation into the factors associated with cardiotoxic events related to CDK4/6 inhibitors using multifactorial logistic regression analysis

Multivariate logistic regression analysis was performed to identify factors associated with cardiovascular adverse event reporting among BC patients receiving CDK4/6 inhibitors. Covariates included age, body weight, treatment duration, sex, inhibitor subtype, and concomitant administration of aromatase inhibitors or fulvestrant.

**FIGURE 7:**
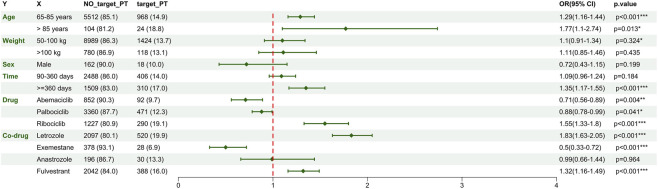
Multivariate logistic regression analysis of the ratios of cardiotoxic events associated with CDK4/6 inhibitors. This forest plot illustrates the adjusted odds ratios and 95% CI for factors influencing the reporting of cardiotoxicity associated with CDK4/6 inhibitors, derived from a multivariate logistic regression model. The analysis was adjusted for age (reference: 65 ≤ AGE <85), sex (reference: male), type of CDK4/6 inhibitor (reference: ribociclib), and specific comorbidities (reference: use of letrozole, anastrozole, exemestane or fulvestrant). Statistical significance is denoted by asterisks: *** (p < 0.001), ** (p < 0.01), and * (p < 0.05). Abbreviations: OR, odds ratios; X, represents the independent variables; Y, represents the dependent variables; 95%CI, the 95% confidence interval.

The key finding was a significantly higher reporting proportion of CVAEs in patients aged 65–85 years (OR = 1.29, 95% CI: 1.16–1.44, p < 0.001). By gender, cardiotoxicity occurred less frequently in men, though this difference was not statistically significant (OR = 1.11, 95% CI: 0.85–1.46, p = 0.199). Among CDK4/6 inhibitors, ribociclib was associated with a significantly higher reporting association with cardiotoxicity (OR = 1.55, 95% CI: 1.31–1.80, p < 0.001) compared to abemaciclib (OR = 0.71) and palbociclib (OR = 0.88). Furthermore, letrozole (OR = 1.83, 95% CI: 1.65–2.05, p < 0.001) or fulvestrant (OR = 1.32, 95% CI: 1.16–1.49, p < 0.001) was associated with increased cardiotoxicity reporting when combined with CDK4/6 inhibitors (Figure 7).

#### 3.3.2 Analysis of the risk of hospitalization and death due to serious adverse events induced by three types of CDK4/6 inhibitors drugs

**FIGURE 8:**
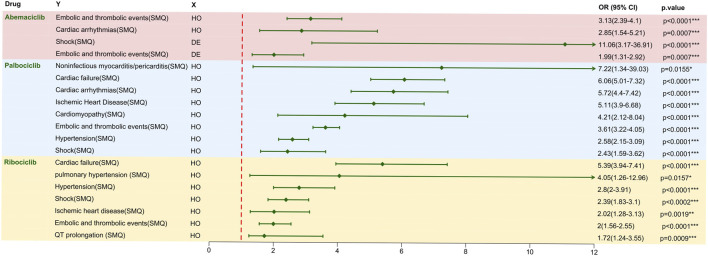
Regression analysis of hospitalization and death related to serious adverse events of CDK4/6 inhibitors. Abbreviations: OR, odds ratios; X, represents the independent variables; Y, represents the dependent variables. HO, hospitalization; DE, death.

Regression analyses were performed to explore reporting associations between SMQs and serious outcomes (hospitalization and death), and to compare these reporting patterns across the three CDK4/6 inhibitors (Figure 8).

For abemaciclib, two SMQs were identified as correlates of increased hospitalization reporting signals: embolic and thrombotic events (OR = 3.13) and cardiac arrhythmias (OR = 2.85). Two SMQs were also associated with fatal outcome reporting: shock (OR = 11.06) and embolic/thrombotic events (OR = 1.99).

Eight SMQs were significantly associated with hospitalization reporting among patients using palbociclib. Sorted by OR values from highest to lowest, these events included noninfectious myocarditis (OR = 7.22), cardiac failure (OR = 6.06), cardiac arrhythmias (OR = 5.72), ischemic heart disease (OR = 5.11), cardiomyopathy (OR = 4.21), embolic/thrombotic events (OR = 3.61), hypertension (OR = 2.58) and shock (OR = 2.43).

For ribociclib, seven SMQs were associated with hospitalization reporting: cardiac failure (OR = 5.39), pulmonary hypertension (OR = 4.05), hypertension (OR = 2.80), shock (OR = 2.39), ischemic heart disease (OR = 2.02), embolic/thrombotic events (OR = 2.00), and QT prolongation (OR = 1.72). The forest plot suggests abemaciclib was linked to fewer hospitalization-related reporting signals relative to the other agents. Across all three inhibitors, univariate analysis identified shock as a significant fatal outcome reporting correlate (p < 0.05).

### 3.4 Onset time and survival analysis of CVAEs in HR+/HER2− BC patients treated with CDK4/6 inhibitors

**FIGURE 9:**
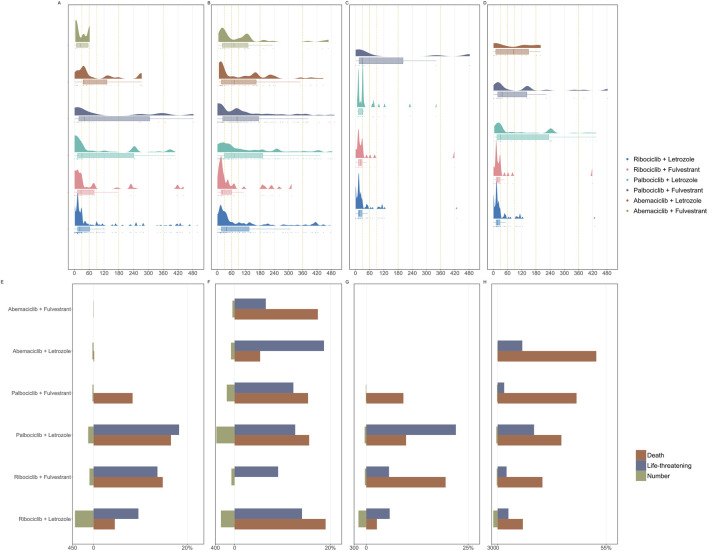
Cloud and rain plots illustrating the onset timing of cardiovascular adverse reactions across distinct combination regimens. **(A,E)** show cardiac arrhythmias; **(B,F)** represent embolic and thrombotic events; **(C,G)** indicate QT prolongation; **(D,H)** correspond to shock. The bar graphs below present outcome indicators for cardiovascular adverse events related to these combination therapies.

We analyzed the reporting timing and clinical outcomes of adverse events across four SMQs for the three CDK4/6 inhibitors. When combined with letrozole, all three agents showed a prominent peak of CVAE reporting within the first 30 days. Thereafter, CVAE reporting frequency decreased gradually and reached its lowest level at 270–480 days. The median time to onset of embolic and thrombotic event reports was 220 days for abemaciclib plus letrozole. For ribociclib combined with fulvestrant, most CVAEs were reported within 90 days, followed by a sustained decline. Notably, arrhythmia reporting rebounded between day 210 and day 240. The median time to onset of shock reports was 142 days in patients receiving palbociclib plus fulvestrant (Figure 9).

Moreover, mortality reporting associated with CVAEs across different regimens was analyzed and illustrated in bar charts (Figure 9). Ribociclib plus fulvestrant was linked to a low mortality reporting frequency for embolic and thrombotic events. Abemaciclib plus letrozole had the highest shock-related mortality reporting rate (n = 8, 50.00%) (Supplementary Table S3).

All covariates retained in the final regression model presented VIF values below 10, demonstrating no obvious multicollinearity. The Hosmer–Lemeshow test yielded a p > 0.05, confirming satisfactory goodness-of-fit of the constructed regression model.

## 4 Discussion

Multiple clinical trials have shown that CDK4/6 inhibitors combined with an AI or fulvestrant significantly prolong PFS, OS, and improve prognosis in HR+/HER2- BC patients (Slamon et al., 2018; Xu et al., 2022; Cristofanilli et al., 2016; Johnston et al., 2019; Goetz et al., 2024; Sledge et al., 2017; Hortobagyi et al., 2016; Hortobagyi et al., 2022; Slamon et al., 2021). This study performs a comprehensive pharmacovigilance analysis based on FAERS to investigate the association, incidence, and outcomes of CVAEs with CDK4/6 inhibitors, comparing abemaciclib, palbociclib, and ribociclib across SMQs. A total of 4,002 CVAEs were analyzed, excluding single drugs in combination therapies to mitigate confounding effects.

### 4.1 Burden and clinical characteristics of CVAEs in HR+/HER2- BC treated with CDK4/6 inhibitors

Among BC patients on CDK4/6 inhibitors, CVAEs constituted 8.13% of all reported adverse events. Females exhibited a significantly higher CVAE rate, which may be explained by the higher prevalence of breast cancer and corresponding drug exposure in women (Britt et al., 2020; Obeagu and Obeagu, 2024). The median patient age was 67 years, with older patients (≥65) showing higher CVAEs risk. Multivariate analysis identified age as a key factor for both BC progression and CVAEs during treatment (Michaels et al., 2023). CDK4/6 inhibitors in combination with letrozole or fulvestrant are commonly used as first-line or second-line treatments for HR+/HER2- BC and increase CVAEs risk, proactive cardiovascular monitoring in elderly patients is crucial.

### 4.2 CVAEs profile of HR+/HER2- BC treated with CDK4/6 inhibitors

Ribociclib was associated with the highest number of CVAEs, including arrhythmias, QT prolongation, and shock in combination with letrozole/fulvestrant, consistent with MONALEESA trials (Tripathy et al., 2018). Notably, we detected no obvious signals of hypertension, which may be attributed to inherent limitations of the database.

Overall, palbociclib had a relatively weak overall CVAEs signal. However, stronger signals for arrhythmia, QT prolongation and shock were detected for this agent relative to abemaciclib and ribociclib. This may relate to its early FDA approval and wider post-market use. PALOMA-4 (Xu et al., 2022) showed the combination of palbociclib and letrozole caused QT prolongation and pulmonary embolism. PALOMA-3 (Cristofanilli et al., 2016) found dyspnoea, hypertension, thromboembolism and QT prolongation in patients on palbociclib plus fulvestrant. To achieve a comprehensive assessment of cardiovascular safety signals, real-world evidence and long-term post-marketing surveillance are indispensable. The inconsistencies observed between real-world and trial data also stress the importance of sustained monitoring in heterogeneous patient cohorts.

The MONARCH-2 trial showed that abemaciclib combined with fulvestrant vs. fulvestrant alone had similar dyspnoea rates but a clinically significant increase in thromboembolic events (2.0% vs. 0.4%) (Sledge et al., 2017). This study found a reporting ROR of 2.4 for thrombotic events with the combination, consistent with other trials. Further screening of PT signals for CDK4/6 inhibitors indicated that thrombosis and embolism accounted for three out of the top five positive signals. Clinicians should therefore strengthen strategies to address thrombotic risks during administration of abemaciclib plus fulvestrant.

### 4.3 Potential mechanisms of CVAEs in HR+/HER2- BC treated with CDK4/6 inhibitors

Findings highlight distinct CVAE profiles among CDK4/6 inhibitors, necessitating individualized cardiovascular monitoring for BC patients. Proposed mechanisms of cardiovascular toxicity include direct effects on vascular inflammation/left ventricular remodeling, PI3K/AKT pathway downregulation, and disruption of potassium/sodium channels (Fradley et al., 2023; Santoni et al., 2019; Abu-Khalaf et al., 2023). Inhibition of Kv11.1 (encoded by KCNH2), a key cardiac potassium channel mediating rapid delayed rectifier current, prolongs QT intervals and increases arrhythmia risk (Li et al., 2025).

### 4.4 Time to adverse reaction induction and associated outcomes in the development of CVAEs in HR+/HER2- BC treated with CDK4/6 inhibitors

The median time to CVAEs was 41–49 days, with incidence decreasing to lowest at 271–360 days but rebounding >360 days, indicating time-varying risk. Embolic events were the main hospitalization risk, while shock strongly correlated with mortality, highlighting the need for timely interventions.

### 4.5 CVAEs and non-CVAEs occur concurrently in HR+/HER2- BC patients treated with CDK4/6 inhibitors

In CDK4/6 inhibitor-treated patients, CVAEs and non-CVAEs co-occur, with haematopoietic cytopenias (neutropenia most common; rapidly reversible due to cytostatic effects (Asghar et al., 2015)), hypersensitivity (rash: 11%–19%, mild-moderate severity (Tripathy et al., 2018; Amigo et al., 2022; Karagounis et al., 2018)), and hyperglycemia/diabetes being predominant non-CVAEs.

Diabetes (T2DM) is a major cardiovascular risk factor: 16% of BC patients <64 years had pre-existing T2DM (Spinetti et al., 2023; Lawrence et al., 2020), while nearly 10% developed it post-treatment (Lipscombe et al., 2013). Shared mechanisms (e.g., obesity, age (Eketunde, 2020)) suggest BC with diabetes may contribute to CVAEs. Clinicians must monitor hematologic/CVAEs toxicity closely and implement early interventions.

### 4.6 Risk assessment and management of cardiovascular toxicity in HR+/HER2- BC treatment

Effective early detection and management of cardiovascular toxicity are critical for optimizing clinical outcomes in BC patients. Especially with the development of novel anticancer agents, there is a growing demand for robust strategies to manage cardiovascular disease during treatment. Leading oncology and cardiology societies-including the European Society for Medical Oncology (ESMO), National Comprehensive Cancer Network (NCCN), American Society of Clinical Oncology (ASCO), European Society of Cardiology (ESC), and Chinese Society of Clinical Oncology (CSCO) have established guidelines for monitoring and managing treatment-related cardiotoxicity (Curigliano et al., 2020; Armenian et al., 2017; Lyon et al., 2022; Committee CSoCOGW, 2023).

Identifying cardiovascular risk factors and pre-existing diseases before cancer treatment, along with careful consideration of individual patient risks, is crucial for reducing adverse cardiovascular events. This facilitates the selection of suitable treatment options and development of necessary cardiac monitoring plans.

During treatment with drugs potentially causing cardiotoxicity, close monitoring of symptoms and signs is recommended. Beyond left ventricular ejection fraction (LVEF) and cardiac biomarkers, global longitudinal strain (GLS) provides enhanced sensitivity for detecting subclinical myocardial dysfunction undetectable by conventional methods (Thavendir et al., 2014).

Notably, some statistically prominent PT signals lacked credible clinical and toxicological plausibility, such as surgical procedures, packaging-related complaints, yawning, lithiasis and improvement of baseline diseases. These irrelevant correlations likely stemmed from random coincidence or inherent limitations of spontaneous reporting data rather than CDK4/6 inhibitor-induced adverse effects; accordingly, we separated these items from clinically meaningful cardiovascular signals when interpreting results.

### 4.7 Limitations

This study exhibits several unavoidable limitations. First, this analysis relied on spontaneous adverse event reports from the FAERS database. Notably, all outcomes only demonstrate disproportional reporting signals and statistical correlations, rather than representing genuine clinical incidence or absolute risk. To overcome this constraint, subsequent real-world studies may incorporate multi-center cohort data with comprehensive patient exposure information to accurately characterize the true incidence of cardiovascular toxicities. Second, different CDK4/6 inhibitors are used in distinct treatment lines and patient populations. In clinical practice, palbociclib, ribociclib, and abemaciclib are prescribed for distinct disease stages, prior therapies, and comorbidity profiles, and endocrine partners also differ by disease status. These systematic differences cannot be adjusted in FAERS, potentially distorting CVAE reporting associations. Future large-scale, real-world cohort studies with detailed clinical covariates are needed to minimize such confounding. Third, spontaneous reporting databases are inherently susceptible to duplicated entries and incomplete case information, which may introduce reporting bias and affect the accuracy of signal detection. Standardized data cleaning protocols and cross-validation across multiple authoritative medical databases in future work can effectively reduce such data-related biases. Fourth, the observed increase in the proportion of CVAE reports from 3% (2015) to 14.7% (2024) should not be interpreted as a true increase in CVAE incidence. This trend is likely driven by increased drug utilization, improved reporting practices, and heightened safety awareness over time, rather than a genuine rise in cardiovascular toxicity. Fifth, partial CVAEs showed inconsistent signal trends between ROR and IC analyses. Such divergence largely stemmed from insufficient case numbers, which rendered the IC algorithm overly conservative and unable to satisfy the conventional IC025 > 0 screening criterion. Accumulating long-term post-marketing surveillance data and expanding the sample size in subsequent research can stabilize signal calculation and unify analytical results. Being an exploratory database analysis, these findings necessitate confirmation in large prospective trials. Future clinical studies are planned to validate results, thereby strengthening pharmacovigilance applications for real-world risk assessment. Finally, this study could not fully exclude the confounding impacts of concurrent cardiotoxic medications and pre-existing cardiovascular comorbidities, making it infeasible to establish a definite causal relationship between CDK4/6 inhibitor administration and cardiovascular toxicities. Well-designed controlled studies with rigorous confounding factor adjustment and stratified analysis are needed to further verify the reliability of these reporting associations.

## 5 Conclusion

CDK4/6 inhibitor combinations show notable cardiovascular reporting signals, with more frequent CVAE reports in letrozole-containing regimens. These signals suggest enhanced vigilance for thrombosis, arrhythmias, QT prolongation, and shock may be warranted. Cohort and long-term trials are needed to validate these safety findings.

## Data Availability

The original contributions presented in the study are included in the article/Supplementary Material, further inquiries can be directed to the corresponding authors.

## Supplementary material

Supplemental Table S1 Cardiovascular adverse events categorization according to Medical Dictionary for Regulatory Activities (MedDRA) Classification Version 26.1.

Supplemental Table S2 List of preferred terms within the standardized MedDRA Query cardiovascular adverse events (cardiac failure, cardiac arrhythmias, cardiomyopathy, embolic and thrombotic events, hypertension, pulmonary hypertension, QT prolongation, noninfectious myocarditis, ischemic heart disease, and shock).

Supplemental Table S3 Records and proportions of death/life-threatening outcomes of cardiovascular adverse events.
