# Supplementary material for: Cardiovascular toxicity of CDK4/6 inhibitors combined with endocrine therapy versus endocrine therapy alone in HR+/HER2- breast cancer: a real-world study based on the FAERS database
Source: Front Pharmacol. 2026 Jul 1;17:1831639. doi: 10.3389/fphar.2026.1831639 (PMC13369606; doi:10.3389/fphar.2026.1831639)
Supplement: Supplementary file 1 [file Table1.DOCX]

Supplemental table S1: Cardiovascular adverse events categorization according to Medical Dictionary for Regulatory Activities (MedDRA) Classification Version 26.1.

| Group | MedDRA terms included |
| --- | --- |
| Heart Failure | Cardiac failure (SMQ narrow 26.1) |
| Hypertension | Hypertension (SMQ narrow 26.1) |
| Ischemic heart disease | Ischaemic heart disease (SMQ broad 26.1) |
| Torsade de pointes/QT prolongation | Torsade de pointes/QT prolongation (SMQ narrow 26.1) |
| Cardiac arrhythmias | Cardiac arrhythmias (SMQ narrow 26.1) |
| Cardiomyopathy | Cardiomyopathy (SMQ narrow 26.1) |
| Venous embolic events | Embolic and thrombotic events (SMQ narrow 26.1) |
| Pulmonary hypertension and related cardiac involvemen | Pulmonary hypertension (SMQ narrow 26.1) |
| Noninfectious myocarditis | Coninfectious myocarditis (SMQ narrow 26.1) |
| Shock | Shock (SMQ narrow 26.1) |

SMQ: Standardized MedDRA Queries.

Supplemental table S2: List of preferred terms within the standardized MedDRA Query cardiovascular adverse events (cardiac failure, cardiac arrhythmias, cardiomyopathy, embolic and thrombotic events, hypertension, pulmonary hypertension, QT prolongation, noninfectious myocarditis, ischemic heart disease, and shock).

| SMQ | PT |
| --- | --- |
| Cardiac failure | Congestive hepatopathy  Ejection fraction decreased  Right ventricular ejection fraction decreased  Radiation associated cardiac failure  Pulmonary oedema  Acute pulmonary oedema  Pulmonary oedema neonatal  Cardiac failure congestive  Low cardiac output syndrome  Cor pulmonale  Hepatojugular reflux  Cardiac failure high output  Cor pulmonale acute  Cardiac failure acute  Acute right ventricular failure  Acute left ventricular failure  Cor pulmonale chronic  Cardiac failure chronic  Chronic right ventricular failure  Chronic left ventricular failure  Neonatal cardiac failure  Cardiopulmonary failure  Cardiohepatic syndrome  Cardiac failure  Cardiorenal syndrome  Ventricular failure  Cardiac asthma  Cardiogenic shock  Right ventricular failure  Left ventricular failure  Obstructive shock |
| Cardiac arrhythmias | Electrocardiogram PR prolongation  Electrocardiogram PR shortened  Electrocardiogram QRS complex prolonged  Electrocardiogram QT prolonged  Electrocardiogram RR interval prolonged  Electrocardiogram RR interval shortened  Electrocardiogram RR interval abnormal  Electrocardiogram U wave inversion  Electrocardiogram U wave present  Electrocardiogram U-wave abnormality  Electrocardiogram delta waves abnormal  Electrocardiogram repolarisation abnormality  Electrocardiogram repolarisation abnormality  Heart rate irregular  Ictal bradycardia syndrome  Andersen-Tawil syndrome  Brugada syndrome  Brugada syndrome  Atrioventricular node dispersion  Familial atrial fibrillation  Familial atrial fibrillation  Lown-Ganong-Levine syndrome  Chronic atrial and intestinal dysrhythmia syndrome  Long QT syndrome congenital  Congenital supraventricular tachycardia  Congenital supraventricular tachycardia  Wolff-Parkinson-White syndrome congenital  Heart block congenital  Arrhythmogenic right ventricular dysplasia  Pacemaker generated arrhythmia  Pacemaker syndrome  Sudden cardiac death  BRASH syndrome  Frederick's syndrome  Adams-Stokes syndrome  Parasystole  Long QT syndrome  Withdrawal arrhythmia  Conduction disorder  Atrioventricular block second degree  Atrial fibrillation  Atrial flutter  Atrioventricular conduction time shortened  Atrioventricular dissociation  Atrioventricular node dysfunction  Atrioventricular block  Atrial parasystole  Atrial tachycardia  Atrial escape rhythm  Atrial escape rhythm  Fascicular block  Bradyarrhythmia  Accelerated idioventricular rhythm  Holiday heart syndrome  Torsade de pointes  Junctional ectopic tachycardia  Junctional ectopic tachycardia  Nodal rhythm  Nodal arrhythmia  Tachyarrhythmia  Lenegre's disease  Agonal rhythm  Extrasystoles  Wandering pacemaker  Trifascicular block  Ventricular fibrillation  Defect conduction intraventricular  Ventricular flutter  Supraventricular tachyarrhythmia  Supraventricular extrasystoles  Supraventricular tachycardia  Arrhythmia supraventricular  Ventricular tachyarrhythmia  Ventricular extrasystoles  Ventricular asystole  Ventricular tachycardia  Ventricular arrhythmia  Bundle branch block  Bifascicular block  Bundle branch block bilateral  Foetal tachyarrhythmia  Foetal arrhythmia  Foetal heart rate disorder  Atrioventricular block complete  Wolff-Parkinson-White syndrome  Pulseless electrical activity  Neonatal bradyarrhythmia  Neonatal tachyarrhythmia  Arrhythmia neonatal  Atrial conduction time prolongation  Atrial standstill  Arrhythmia  Arrhythmic storm  Ventricular dyssynchrony  Ventricular parasystole  Ventricular pre-excitation  Rhythm idioventricular  Chronotropic incompetence  Cardiac fibrillation  Cardiac fibrillation  Heart alternation  Accessory cardiac pathway  Cardiac flutter  Atrioventricular block first degree  Anomalous atrioventricular excitation  Ectopic atrial rhythm  Ectopic atrial rhythm  Bundle branch block right  Reperfusion arrhythmia  Early repolarisation syndrome  Early repolarisation syndrome  Paroxysmal atrioventricular block  Paroxysmal arrhythmia  Bundle branch block left  Sinus node dysfunction  Sinoatrial block  Sinus arrest  Sinus bradycardia  Sinus tachycardia  Sinus arrhythmia |
| Cardiomyopathy | Chagas cardiomyopathy  HIV cardiomyopathy  Viral cardiomyopathy  Septic cardiomyopathy  Pulmonary arterial wedge pressure increased  Ejection fraction decreased  Ejection fraction abnormal  Biopsy heart abnormal  Right ventricular ejection fraction decreased  Hypertrophic cardiomyopathy  Muscular dystrophy  Kearns-Sayre syndrome  Mitochondrial cardiomyopathy  Peripartum cardiomyopathy  Metabolic cardiomyopathy  Non-obstructive cardiomyopathy  Obesity cardiomyopathy  Hypertensive cardiomyopathy  Atrial septal defect acquired  Ventricular septal defect acquired  Cardiomyopathy acute  Thyrotoxic cardiomyopathy  Cardiomyopathy alcoholic  Giant cell myocarditis  Dilated cardiomyopathy  Uraemic cardiomyopathy  Pacing induced cardiomyopathy  Ischaemic cardiomyopathy  Eosinophilic myocarditis  Diabetic cardiomyopathy  Restrictive cardiomyopathy  Cardiomyopathy neonatal  Tachycardia induced cardiomyopathy  Cardiomyopathy  Myocardial haemorrhage  Myocardial calcification  Myocardial fibrosis  Cardiac amyloidosis  Cardiotoxicity  Cardiac hypertrophy  Cardiac septal hypertrophy  Cardiac sarcoidosis  Cardiac iron overload  Stress cardiomyopathy  Toxic cardiomyopathy |
| Embolic and thrombotic events | Thrombosis in device  Device occlusion  Inner ear infarction  Budd-Chiari syndrome  Hepatic artery embolism  Hepatic artery thrombosis  Hepatic infarction  Hepatic vein occlusion  Hepatic vein embolism  Hepatic vein thrombosis  Hepatic vascular thrombosis  Hepatic artery occlusion  Venoocclusive liver disease  Portosplenomesenteric venous thrombosis  Portal vein occlusion  Portal shunt  Portal vein cavernous transformation  Portal vein embolism  Portal vein thrombosis  Cavernous sinus thrombosis  Cerebral septic infarct  Septic pulmonary embolism  Embolic pneumonia  SI QIII TIII pattern  Ultrasonic angiogram abnormal  Directional Doppler flow tests abnormal  Arteriogram abnormal  Ultrasound Doppler abnormal  Homans' sign positive  Arteriogram carotid abnormal  Venogram abnormal  Angiogram cerebral abnormal  Angiogram abnormal  Fluorescence angiogram abnormal  Angiogram peripheral abnormal  Aortogram abnormal  Haemorrhagic cerebral infarction  Haemorrhagic stroke  Cerebral congestion  Cerebral artery occlusion  Cerebral artery embolism  Cerebral artery thrombosis  Cerebral infarction  Cerebral venous sinus thrombosis  Precerebral artery thrombosis  Cerebral ischaemia  Cerebral microinfarction  Middle cerebral artery stroke  Metabolic stroke  Hemiparesis  Monoplegia  Monoparesis  Parietal lobe stroke  Transient ischaemic attack  Transverse sinus thrombosis  Basilar artery occlusion  Basilar artery thrombosis  Basal ganglia infarction  Basal ganglia stroke  Spinal artery embolism  Spinal artery thrombosis  Spinal cord infarction  Spinal stroke  Paraplegia  Stroke in evolution  Stroke in evolution  Carotid artery occlusion  Carotid arterial embolus  Carotid artery thrombosis  Pseudo-occlusion of internal carotid artery  Brain stem infarction  Brain stem embolism  Brain stem thrombosis  Brain stem stroke  Cerebral hypoperfusion  Cerebrospinal thrombotic tamponade  Cerebral venous thrombosis  Cerebral microembolism  Cerebral vascular occlusion  Cerebral vascular occlusion  Cerebrovascular insufficiency  Cerebrovascular stenosis  Cerebrovascular accident  Cerebrovascular disorder  Cerebral thrombosis  Internal capsule infarction  Capsular warning syndrome  Hemiplegia  Lacunar infarction  Paresis  Thalamic infarction  Thalamic stroke  Ischaemic cerebral infarction  Ischaemic stroke  Precerebral artery occlusion  Precerebral artery embolism  Superior sagittal sinus thrombosis  Visual midline shift syndrome  Embolic cerebral infarction  Embolic stroke  Diplegia  Quadriparesis  Quadriplegia  Cerebral infarction foetal  Foetal cerebrovascular disorder  Aseptic cavernous sinus thrombosis  Paraparesis  Cerebellar artery occlusion  Cerebellar artery thrombosis  Cerebellar infarction  Cerebellar embolism  Cerebellar embolism  Thrombotic cerebral infarction  Embolic cerebellar infarction  Thrombotic stroke  Sigmoid sinus thrombosis  Occipital lobe stroke  Vertebral artery occlusion  Vertebral artery thrombosis  Vertebrobasilar infarction  Haemorrhagic transformation stroke  Post procedural pulmonary embolism  Post procedural myocardial infarction  Post procedural stroke  Arteriovenous graft thrombosis  Arteriovenous fistula occlusion  Arteriovenous fistula thrombosis  Pulmonary oil microembolism  Shunt thrombosis  Shunt occlusion  Coronary bypass thrombosis  Coronary vascular graft occlusion  Coronary artery reocclusion  Incision site vessel occlusion  Postoperative thrombosis  Deep vein thrombosis postoperative  Intraoperative cerebral artery occlusion  Peripheral arterial reocclusion  Incomplete atrial appendage closure  Vascular access site thrombosis  Vascular pseudoaneurysm thrombosis  Vascular graft occlusion  Vascular graft thrombosis  Graft thrombosis  Stoma site thrombosis  Bone infarction  Muscle infarction  Mesenteric artery stent insertion  Cerebral angioplasty  Catheter directed thrombolysis  Arterial bypass operation  Endarterectomy  Arterectomy with graft replacement  Arterectomy  Arteriotomy  Arterial angioplasty  Arterial revascularisation  Arterial graft  Arterial recanalisation procedure  Arterial stent insertion  Arterial therapeutic procedure  Pulmonary endarterectomy  Pulmonary artery stent insertion  Pulmonary artery therapeutic procedure  Pulmonary angioplasty  Profundaplasty  Coronary arterial stent insertion  Coronary artery bypass  Coronary endarterectomy  Coronary artery surgery  Coronary angioplasty  Coronary angioplasty  Coronary revascularisation  Percutaneous coronary intervention  Atherectomy  Carotid angioplasty  Carotid artery bypass  Carotid endarterectomy  Carotid artery stent insertion  Carotid revascularisation  Catheterisation venous  Phlebectomy  Venous operation  Venous repair  Venous angioplasty  Venous stent insertion  Portal shunt procedure  Cerebral artery stent insertion  Cerebral bypass surgery  Cerebrovascular operation  Cerebrovascular accident prophylaxis  Cerebral revascularisation  Pneumatic compression therapy  Vena cava filter removal  Vena cava filter insertion  Thrombolysis  Renal artery angioplasty  Renal artery angioplasty  Renal artery revascularisation  Surgical vascular shunt  Peripheral artery bypass  Peripheral endarterectomy  Peripheral artery surgery  Peripheral artery angioplasty  Peripheral artery stent insertion  Peripheral revascularisation  Atrial appendage closure  Atrial appendage resection  Angioplasty  Angioplasty  Prosthetic vessel implantation  Vasodilation procedure  Vascular operation  Vascular graft  Vascular graft  Angiostomy  Vascular stent insertion  Thrombectomy  Thromboembolectomy  Thrombosis prophylaxis  Revascularisation procedure  Compression garment application  Central venous catheterisation  Tumour thrombectomy  Aortic bypass  Intra-aortic balloon placement  Aortic surgery  Strokectomy  Left atrial appendage closure implant  Obstetrical pulmonary embolism  Pulmonary artery occlusion  Pulmonary artery thrombosis  Pulmonary infarction  Pulmonary vein occlusion  Pulmonary veno-occlusive disease  Pulmonary venous thrombosis  Pulmonary embolism  Pulmonary microemboli  Pulmonary thrombosis  Pulmonary tumour thrombotic microangiopathy  Tumour embolism  Tumour thrombosis  Pituitary infarction  Thyroid infarction  Haemorrhagic adrenal infarction  Adrenal thrombosis  Catheter site thrombosis  Instillation site thrombosis  Administration site thrombosis  Device related thrombosis  Device embolisation  Artificial blood vessel occlusion  Infusion site thrombosis  Application site thrombosis  Prosthetic cardiac valve thrombosis  Vessel puncture site occlusion  Vessel puncture site thrombosis  Vascular stent occlusion  Vascular stent thrombosis  Vascular device occlusion  Embolia cutis medicamentosa  Medical device site thrombosis  Vaccination site thrombosis  Implant site thrombosis  Injection site thrombosis  Umbilical cord occlusion  Umbilical cord thrombosis  Foetal vascular malperfusion  Placental infarction  Renal artery thrombosis  Renal artery occlusion  Renal infarct  Renal vein embolism  Renal vein thrombosis  Renal vein occlusion  Renal-limited thrombotic microangiopathy  Renal embolism  Renal vascular thrombosis  Spermatic vein thrombosis  Ovarian vein thrombosis  Penile artery occlusion  Thrombosis corpora cavernosa  Penile vein thrombosis  Testicular infarction  Intestinal infarction  Mesenteric arterial occlusion  Mesenteric arterial occlusion  Mesenteric artery embolism  Mesenteric artery stenosis  Mesenteric artery thrombosis  Mesenteric arteriosclerosis  Mesenteric venous occlusion  Mesenteric venous occlusion  Mesenteric vein embolism  Mesenteric vein thrombosis  Superior mesenteric artery syndrome  Mesenteric vascular occlusion  Mesenteric vascular insufficiency  Thrombosis mesenteric vessel  Truncus coeliacus thrombosis  Visceral venous thrombosis  Gastric infarction  Haemorrhoids thrombosed  Pancreatic infarction  Mahler sign  Coronary artery occlusion  Coronary artery embolism  Coronary artery thrombosis  Coronary artery thrombosis  Acute coronary syndrome  Acute myocardial infarction  Lambl's excrescences  Papillary muscle infarction  Tricuspid valve thrombosis  Silent myocardial infarction  Silent myocardial infarction  Atrial thrombosis  Myocardial infarction  Postinfarction angina  Myocardial necrosis  Intracardiac thrombus  Intracardiac mass  Cardiac ventricular thrombosis  Stress cardiomyopathy  May-Thurner syndrome  Thromboangiitis obliterans  Collateral circulation  Postpartum venous thrombosis  Postpartum thrombosis  Haemorrhagic infarction  Arterial occlusive disease  Aneurysm thrombosis  Embolism arterial  Arterial thrombosis  Atherosclerotic plaque rupture  Paradoxical embolism  Paraneoplastic thrombosis  Coeliac artery occlusion  Infarction  Femoral artery embolism  Acute aortic syndrome  Segmental arterial mediolysis  Jugular vein occlusion  Jugular vein embolism  Jugular vein thrombosis  Venous occlusion  Venoocclusive disease  Embolism venous  Venous thrombosis  Venous recanalisation  Leriche syndrome  Popliteal artery entrapment syndrome  Paget-Schroetter syndrome  Pelvic venous thrombosis  Superficial vein thrombosis  Vena cava embolism  Vena cava thrombosis  Venous thrombosis in pregnancy  Superior vena cava occlusion  Superior vena cava syndrome  Deep vein thrombosis  Embolism  Embolism  Venous thrombosis limb  Subclavian artery occlusion  Subclavian artery embolism  Subclavian artery thrombosis  Subclavian vein occlusion  Subclavian vein embolism  Subclavian vein thrombosis  Brachiocephalic artery occlusion  Brachiocephalic vein occlusion  Brachiocephalic vein thrombosis  Peripheral artery occlusion  Peripheral arterial occlusive disease  Peripheral artery thrombosis  Peripheral vein occlusion  Peripheral vein thrombosis  Peripheral vein thrombus extension  Peripheral embolism  Microembolism  Inferior vena caval occlusion  Inferior vena cava syndrome  Hypothenar hammer syndrome  Venous thrombosis neonatal  Thrombophlebitis neonatal  Thrombosis  Post thrombotic syndrome  Thrombosed varicose vein  Thrombophlebitis  Axillary vein thrombosis  Thrombophlebitis migrans  Aortic aneurysm thrombosis  Aortic embolus  Aortic thrombosis  Obstructive shock  Iliac artery occlusion  Iliac artery embolism  Iliac vein occlusion  Heparin-induced thrombocytopenia  Antiphospholipid syndrome  Disseminated intravascular coagulation  Splenic artery thrombosis  Splenic infarction  Splenic vein occlusion  Splenic vein thrombosis  Splenic embolism  Splenic thrombosis  Disseminated intravascular coagulation in newborn  Thrombosis with thrombocytopenia syndrome  Thrombotic microangiopathy  Thrombotic thrombocytopenic purpura  Spontaneous heparin-induced thrombocytopenia syndrome  Autoimmune heparin-induced thrombocytopenia  Amaurosis  Choroidal infarction  Visual acuity reduced transiently  Optic nerve infarction  Retinal artery occlusion  Retinal artery embolism  Retinal artery thrombosis  Retinal infarction  Retinal vein occlusion  Retinal vein thrombosis  Retinal vascular thrombosis  Ophthalmic artery occlusion  Ophthalmic artery thrombosis  Eye infarction  Ophthalmic vein thrombosis  Ophthalmic vascular thrombosis  Amaurosis fugax  Blindness transient |
| Hypertension | Metabolic syndrome  Blood pressure ambulatory increased  Mean arterial pressure increased  Blood pressure systolic increased  Blood pressure diastolic increased  Blood pressure increased  Blood pressure orthostatic increased  Hypertensive encephalopathy  Hypertensive cerebrovascular disease  Procedural hypertension  Postoperative hypertension  Renal artery revascularisation  Renal sympathetic nerve ablation  Blood pressure management  Syndrome Z  Secondary aldosteronism  Hyperaldosteronism  Primary hyperaldosteronism  Superimposed pre-eclampsia  Gestational hypertension  HELLP syndrome  Pre-eclampsia  Eclampsia  Page kidney  Malignant renal hypertension  Hypertensive nephropathy  Renal hypertension  Malignant hypertensive heart disease  Hypertensive cardiomegaly  Hypertensive cardiomyopathy  Hypertensive heart disease  White coat hypertension  Labile hypertension  Malignant hypertension  Catecholamine crisis  Hypertension  Hypertensive emergency  Prehypertension  Hypertensive crisis  Hypertensive end-organ damage  Hypertensive angiopathy  Hypertensive urgency  Accelerated hypertension  Secondary hypertension  Endocrine hypertension  Neurogenic hypertension  Renovascular hypertension  Systolic hypertension  Diastolic hypertension  Withdrawal hypertension  Dialysis induced hypertension  Hypertension neonatal  Blood pressure inadequately controlled  Supine hypertension  Nocturnal hypertension  Maternal hypertension affecting foetus  Essential hypertension  Orthostatic hypertension  Retinopathy hypertensive |
| Pulmonary hypertension | Pulmonary arterial wedge pressure increased  Pulmonary arterial pressure increased  Pulmonary arterial pressure abnormal  Vascular resistance pulmonary increased  Right ventricular systolic pressure increased  Right atrial volume increased  Right atrial pressure increased  Right ventricular ejection fraction decreased  Cardiac ventriculogram right abnormal  Central venous pressure increased  Pulmonary endarterectomy  Pulmonary artery stent insertion  Pulmonary angioplasty  Pulmonary artery wall hypertrophy  Pulmonary arterial hypertension  Pulmonary artery dissection  Pulmonary artery dilatation  Pulmonary artery thrombosis  Pulmonary vein occlusion  Pulmonary veno-occlusive disease  Pulmonary venous hypertension  Pulmonary vein stenosis  Pulmonary capillary haemangiomatosis  Pulmonary hypertension  Pulmonary hypertensive crisis  Pulmonary vascular resistance abnormality  Portopulmonary hypertension  Neonatal pulmonary hypertension  Pulmonary tumour thrombotic microangiopathy  Pulmonary valve incompetence  Cor pulmonale  Coronary sinus dilatation  Cor pulmonale acute  Acute right ventricular failure  Cor pulmonale chronic  Chronic right ventricular failure  Tricuspid valve incompetence  Right ventricular enlargement  Right ventricular heave  Right atrial hypertrophy  Right atrial enlargement  Right atrial dilatation  Right ventricular hypertrophy  Right ventricular hypertension  Right ventricular dysfunction  Right ventricular dilatation  Right ventricular failure |
| Torsade de pointes/QT prolongation | Electrocardiogram QT prolonged  Electrocardiogram QT interval abnormal  Long QT syndrome congenital  Long QT syndrome  Torsade de pointes  Ventricular tachycardia |
| Noninfectious myocarditis peric | Hypersensitivity myocarditis  Giant cell myocarditis  Chronic myocarditis  Immune-mediated pericarditis  Immune-mediated myocarditis  Eosinophilic myocarditis  Pericarditis constrictive  Pericarditis  Myopericarditis  Myocarditis  Carditis  Pleuropericarditis  Pericarditis adhesive  Autoimmune pericarditis  Autoimmune myocarditis |
| Ischaemic heart disease | Troponin I increased  Troponin T increased  Troponin increased  ECG signs of myocardial ischaemia  Heart-type fatty acid-binding protein increased  Blood creatine phosphokinase MB increased  Blood creatine phosphokinase MB abnormal  Post procedural myocardial infarction  Coronary bypass stenosis  Coronary bypass thrombosis  Coronary vascular graft occlusion  Coronary vascular graft stenosis  Coronary artery reocclusion  Coronary artery restenosis  Periprocedural myocardial infarction  Coronary arterial stent insertion  Coronary artery bypass  Coronary brachytherapy  Coronary endarterectomy  Coronary artery surgery  Coronary angioplasty  Coronary revascularisation  Percutaneous coronary intervention  External counterpulsation  Wellens' syndrome  Prinzmetal angina  Angina unstable  Angina unstable  Coronary artery occlusion  Haemorrhage coronary artery  Coronary artery insufficiency  Coronary artery disease  Coronary artery dissection  Arteriospasm coronary  Coronary ostial stenosis  Coronary steal syndrome  Coronary artery embolism  Coronary no-reflow phenomenon  Coronary artery stenosis  Coronary artery thrombosis  Coronary artery compression  Arteriosclerosis coronary artery  Kounis syndrome  Acute coronary syndrome  Acute myocardial infarction  Acute cardiac event  Acute cardiac event  Chronic coronary syndrome  Ischaemic mitral regurgitation  Ischaemic cardiomyopathy  Papillary muscle infarction  Subclavian coronary steal syndrome  Diabetic coronary microangiopathy  Microvascular coronary artery disease  Silent myocardial infarction  Myocardial stunning  Myocardial infarction  Postinfarction angina  Myocardial hypoperfusion  Cardiac perfusion defect  Myocardial necrosis  Myocardial ischaemia  Myocardial hypoxia  Myocardial reperfusion injury  Angina pectoris  Anginal equivalent  Subendocardial ischaemia  Stress cardiomyopathy |
| Shock | Pulse absent  Electrocardiogram QT prolonged  Electrocardiogram QT interval abnormal  Procedural shock  Long QT syndrome congenital  Cardiac death  Sudden cardiac death  Adams-Stokes syndrome  Long QT syndrome  Atrial parasystole  Acute left ventricular failure  Torsade de pointes  Ventricular fibrillation  Ventricular flutter  Ventricular tachyarrhythmia  Ventricular asystole  Ventricular tachycardia  Ventricular tachycardia  Pulseless electrical activity  Cardio-respiratory arrest neonatal  Cardiac arrest neonatal  Ventricular parasystole  Cardiovascular insufficiency  Cardiogenic shock  Cardiac fibrillation  Cardio-respiratory arrest  Cardiac flutter  Cardiac arrest  Shock  Shock symptom  Circulatory collapse  Circulatory collapse  Obstructive shock |

SMQ: Standardized MedDRA Queries. PT: preferred terms.

Supplemental table S3: Records and proportions of death/life-threatening outcomes of cardiovascular adverse events.

| SMQ | Drug | Number | Death (%) | Life-threatening (%) |
| --- | --- | --- | --- | --- |
| Cardiac arrhythmias | Ribociclib + Letrozole | 397 | 4.53 | 9.57 |
|  | Ribociclib + Fulvestrant | 88 | 14.77 | 13.64 |
|  | Palbociclib + Letrozole | 115 | 16.52 | 18.26 |
|  | Palbociclib + Fulvestrant | 24 | 8.33 | 0 |
|  | Abemaciclib + Letrozole | 25 | 0.12 | 0 |
|  | Abemaciclib + Fulvestrant | 6 | 0 | 0 |
| Embolic and thrombotic events | Ribociclib + Letrozole | 284 | 19.01 | 14.08 |
|  | Ribociclib + Fulvestrant | 66 | 31.82 | 9.09 |
|  | Palbociclib + Letrozole | 379 | 15.57 | 12.66 |
|  | Palbociclib + Fulvestrant | 163 | 15.34 | 12.27 |
|  | Abemaciclib + Letrozole | 75 | 5.33 | 18.67 |
|  | Abemaciclib + Fulvestrant | 46 | 17.39 | 6.52 |
| QT prolongation | Ribociclib + Letrozole | 192 | 2.6 | 5.73 |
|  | Ribociclib + Fulvestrant | 36 | 19.44 | 5.56 |
|  | Palbociclib + Letrozole | 41 | 9.75 | 21.95 |
|  | Palbociclib + Fulvestrant | 11 | 9.09 | 0 |
|  | Abemaciclib + Letrozole | 0 | 0 | 0 |
|  | Abemaciclib + Fulvestrant | 0 | 0 | 0 |
| Shock | Ribociclib + Letrozole | 219 | 12.79 | 5.48 |
|  | Ribociclib + Fulvestrant | 44 | 22.72 | 4.55 |
|  | Palbociclib + Letrozole | 65 | 32.31 | 18.46 |
|  | Palbociclib + Fulvestrant | 30 | 40 | 3.33 |
|  | Abemaciclib + Letrozole | 8 | 50 | 12.5 |
|  | Abemaciclib + Fulvestrant | 0 | 0 | 0 |

SMQ: Standardized MedDRA Queries.
